# Home-based transcranial static magnetic field stimulation − concept, development, and clinical applications: IFCN handbook chapter

**DOI:** 10.1016/j.cnp.2026.04.001

**Published:** 2026-04-09

**Authors:** Claudia Ammann, Vanesa Soto-León

**Affiliations:** a HM CINAC (Centro Integral de Neurociencias Abarca Campal), Hospital Universitario HM Puerta del Sur, HM Hospitales, Madrid, Spain; b Facultad HM de Ciencias de la Salud de la Universidad Camilo José Cela, Madrid, Spain; c Instituto de Investigación Sanitaria HM Hospitales, Spain; d FENNSI Group, Hospital Nacional de Parapléjicos, SESCAM, 45004 Toledo, Spain; e Instituto de Investigación Sanitaria de Castilla-La Mancha (IDISCAM), Carretera Finca la Peraleda, s/n, 45071 Toledo, Spain

**Keywords:** Transcranial static magnetic stimulation, Non-invasive brain stimulation, Home-based neuromodulation, Cortical excitability, Movement disorders, Disease-modifying therapy

## Abstract

Non-invasive brain stimulation (NIBS) techniques, such as transcranial magnetic stimulation (TMS) and transcranial direct current stimulation (tDCS), have significantly advanced neuroscience research and clinical practice. However, these approaches can involve costly equipment (particularly TMS) and varying degrees of technical complexity, especially for protocols requiring precise targeting. Transcranial static magnetic field stimulation (tSMS) has recently emerged as a novel, accessible alternative. This technique uses a simple, constant magnetic field—typically generated by a neodymium magnet—to modulate cortical excitability without electrical input, or perceptible sensation. While static magnets have long been associated with pseudoscientific practices, rigorous studies since 2011 have established the physiological effects of tSMS, demonstrating its capacity to transiently reduce cortical excitability in humans. The simplicity, portability, and excellent safety profile of tSMS make it especially well-suited for home-based applications and long-duration protocols. Although its modulatory effects tend to be modest and transient, recent research indicates that longer stimulation durations can extend the effects, and its predominantly inhibitory action may be advantageous for specific clinical targets. While the static nature of the magnetic field limits depth penetration, ongoing innovations may help address this constraint. Overall, tSMS represents a low-cost, well-tolerated, and practical addition to the NIBS toolkit, with growing potential to support individualized neuromodulation strategies in both research and therapeutic settings.

## Highlights

• Static magnetic stimulation is simple, safe, and reduces cortical excitability.
• Its properties support long-duration and home-based use.
• Clinical studies support its feasibility and translation to clinical practice.

## 1 Introduction

Transcranial static magnetic field stimulation (tSMS) uses a stable magnetic field generated by a permanent magnet to influence cortical excitability (Oliviero et al., 2011), avoiding the need for electronics or external power. Despite its simplicity, tSMS has demonstrated reproducible inhibitory effects over the human motor cortex (Foffani and Oliviero, 2021, Paulus, 2011) and offers advantages in terms of portability, safety, and potential for home-based use (Dileone et al., 2022, Di Lazzaro et al., 2024, 2021).

Magnets have held a place in traditional healing systems for centuries, often with little scientific support. However, modern research has shown that static magnetic fields—when applied with sufficient strength and precision—can influence biological systems (Albuquerque et al., 2016, Rosen, 2003a, Zhang et al., 2023).

Techniques such as transcranial magnetic stimulation (TMS) or transcranial direct current stimulation (tDCS) rely on time-varying magnetic fields or weak direct electrical currents, respectively, whereas tSMS employs a constant magnetic field to subtly alter neural excitability. The method gained recognition following a pivotal study that demonstrated how placing a neodymium magnet over the scalp could transiently reduce motor cortex excitability in humans (Oliviero et al., 2011). Since then, a growing body of evidence has confirmed the reproducibility of its effects across different laboratories and stimulation sites (Foffani and Oliviero, 2021).

**Fig. 1:**
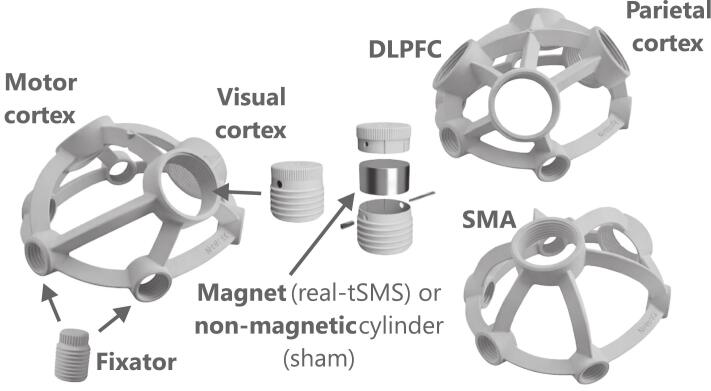
Magnet fixation and ergonomic helmets for tSMS application. Courtesy of Neurek SL, https://www.neurek.com/.

The appeal of tSMS lies in both its biological efficacy and practicality. It is safe, silent, inexpensive, and requires no powered components, complex machinery, or technical expertise (Fig. 1*)*. These features make it a strong candidate for broader clinical use, especially in home-based or long-duration interventions. While its exact mechanisms remain under investigation, tSMS likely modulates ionic movement across neuronal membranes and may also influence glial activity, membrane proteins, or local blood flow (see Section 2.b. for more details).

Though clinical evidence remains limited at this stage, the combination of safety, simplicity, and growing experimental support has spurred interest in translating tSMS from the lab to therapeutic settings. This chapter explores the mechanisms, physiological and behavioral effects, current and future clinical potential and development of tSMS.

## 2 Concept of tSMS

### 2.1 Basic principles of tSMS

tSMS uses permanent neodymium (NdFeB) magnets—compact rare-earth devices known for high magnetic strength, stability, and resistance to demagnetization (Croat et al., 1984). Their reproducible field strength across magnets of the same size (Rivadulla et al., 2014), makes them ideal for standardized research and clinical use.

Unlike other techniques that induce electrical currents, tSMS delivers a static magnetic flux density (B-field) without power supplies or moving parts. The magnet is typically placed with its magnetization axis perpendicular to the scalp to direct the field toward superficial cortex areas (Tharayil et al., 2018). A key advantage is that the magnetic field is largely unaffected by tissue magnetic susceptibility. Simulations show that B-field strength and distribution depend mainly on the magnet’s characteristics and distance to the cortex, making tSMS more predictable and reproducible across individuals (Tharayil et al., 2018). Thus, the strength and gradient of the magnetic field are determined by the magnet’s size, shape, material grade, and placement. A standard N45 magnet (45 mm diameter × 30 mm height) produces ∼ 160–170 mT at 2 cm from the surface—about the average scalp-to-cortex distance in adults—sufficient to modulate cortical excitability (Oliviero et al., 2011). Tharayil et al. (2018) simulated the field distribution of a stronger N52 magnet (5.08 × 2.54 cm) and found cortical B-fields ranging from 160 to 245 mT and gradients from 13.3 to 19.0 T/m, depending on scalp-to-cortex distance and cortical target. These metrics provide critical guidance for optimizing tSMS dosage and positioning. Further modeling by Park et al. (2019) showed that while larger magnets increase flux density, spatial gradients are more shape-dependent. For example, a 60 × 30 mm magnet produced the highest B-field at the cortical surface, whereas a 45 × 30 mm magnet induced the strongest spatial gradient. These findings support the use of mid-sized magnets when focal stimulation is desired, emphasizing the importance of magnet geometry in optimizing tSMS protocols.

Overall, the physical principles underlying tSMS—its constant, predictable magnetic field and independence from tissue-specific physical properties—make it a uniquely simple and promising method for non-invasive neuromodulation.

### 2.2 Mechanisms of action at a physiological and cellular level

Although tSMS is increasingly used in experimental and clinical settings, its physiological mechanisms remain only partially understood. Most human studies have focused on system-level effects such as changes in cortical excitability or behavior, without directly probing underlying cellular or molecular processes. However, a substantial body of *in vitro* and animal research has helped elucidate how static magnetic fields may influence neural activity.

Consistently, animal studies showed that static magnetic fields can suppress neuronal excitability and alter behavior (Ammari et al., 2008, Coots et al., 2004, Houpt et al., 2007, László et al., 2007, Nakagawa and Matsuda, 1988, Nikolić et al., 2008, Okano et al., 2012, Sándor et al., 2007, Yang et al., 2011). For instance, Aguila et al. (2016) demonstrated that applying tSMS over the visual cortex in awake primates impaired visual detection performance, while recordings in anesthetized cats showed a marked reduction in neuronal firing. These findings support the notion that tSMS can exert a direct inhibitory effect on cortical neurons, potentially underlying neurophysiological effects observed in humans (Oliviero et al., 2011).

At the cellular level, a leading hypothesis is that static magnetic fields interact with neuronal membranes by reorienting anisotropically diamagnetic phospholipids, which may affect the conformation and function of embedded ion channels (Rosen, 2003a). Supporting this idea, *in vitro* studies have shown that static fields can slow the activation of voltage-gated Ca^2+^ and Na^+^ channels and influence K^+^ channel inactivation (Rosen, 2003b, Rosen, 1996, Shen et al., 2007). These effects emerge within minutes and are reversible—paralleling the short-term neuromodulation seen in tSMS studies (Rosen and Lubowsky, 1987).

Early work by Azanza and del Moral (1994) in snail neurons further supported this model, showing that static fields (3 to 700 mT) could inhibit activity via Ca^2+^-dependent K^+^ channels. They proposed that magnetic field exposure mobilizes calcium from membrane stores, increasing intracellular Ca^2+^ and boosting K^+^ conductance. At a biophysical level, this may stem from the reorientation of membrane phospholipids (Hughes et al., 2005, Maret and Dransfeld, 1977, Tenforde and Liburdy, 1988), possibly via changes in membrane thickness or ionic interactions (Azanza and del Moral, 1994).

More recent evidence points to Cl^−^ channels as key mediators of tSMS effects. Sinha et al. (2024) found that static magnetic fields increase Cl^−^ conductance through SLC26A11-channels—a non-synaptic anion transporter—producing shunting inhibition and reduced neuronal firing, independent of GABA-A receptor activity. These effects were accompanied by transient neuronal swelling, with minimal reversible changes in Na^+^ or K^+^ currents, suggesting Cl^−^ conductance plays a central role in mediating tSMS effects. Another complementary hypothesis involves magnetic pressure effects on mechanosensitive ion channels. Finite element modeling and empirical data indicated that tSMS magnets generate field gradients and tissue-level forces that, although below activation thresholds, may still influence ion channel gating and local excitability in a reversible, region-specific way, providing a complementary route of tSMS action (Hernando et al., 2020).

While short-term changes may result from reversible membrane changes, longer-lasting after-effects—such as those seen after 30-minute tSMS protocols (Dileone et al., 2018)—may involve sustained cellular plasticity. In this context, static magnetic fields have been shown to modulate NMDA receptor function, alter gene expression linked to synaptic plasticity (Hirai et al., 2002, Hirai and Yoneda, 2005, Hirai and Yoneda, 2004), modify microtubule-associated protein 2 expression and activation of protein degradation pathways (Hirai et al., 2006). Earlier work also reported increased activity of transcription factors such as activator protein 1 (Hirai et al., 2006).

Emerging evidence suggests tSMS also affects glial cells. Early studies reported morphological changes in astrocytes, microglia, and oligodendrocytes after prolonged exposure (Zhadin, 2001), and glial removal in snail models abolished neuronal effects of static fields (Balaban et al., 1990), pointing to an active glial role. Prasad et al. (2017) showed that exposing human oligodendrocyte precursor cells to a 300 mT field increased calcium influx, upregulated L-type calcium channel subunits (CaV1.2, CaV1.3), and promoted neurotrophic factor release. Given the role of astrocytes in plasticity and signaling (Bazargani and Attwell, 2016, Perea and Araque, 1979), their involvement in tSMS effects is plausible. Another proposed mechanism involves magnetite nanoparticles naturally present in the human brain (Dobson and Pierre, 1996, Kirschvink et al., 1992), which strongly interact with magnetic fields and may amplify gradients that influence nearby cells—potentially explaining how weak static fields exert measurable effects.

tSMS may also influence cerebral blood flow. Kim et al. (2010) reported increased perfusion beneath the magnet and decreased perfusion contralateral after 20-minute tSMS in healthy participants. These vascular effects may reflect compensatory physiological responses to neural or glial modulation, even though magnetic fields are expected to reduce perfusion by increasing blood viscosity (Yamamoto et al., 2004). Additionally, it has been hypothesized that slow brain pulsations could generate weak local electric currents when combined with static magnetic fields (Bestmann, 2011, Silva et al., 2006), though this remains unverified.

Finally, the steep magnetic field gradients generated by tSMS may be more biologically effective than uniform fields used in MRI (Tharayil et al., 2018). Several studies suggest that these gradients—rather than absolute field strength—are critical for modulating neural activity (Cavopol et al., 1995, McLean et al., 1995).

In summary, current evidence supports a multifactorial model in which tSMS modulates cortical excitability via ion channel modulation, membrane biophysics, glial activity, gene expression, vascular responses, and gradient-dependent field characteristics. Together, these mechanisms help explain how a simple static magnet can induce measurable and sometimes lasting changes in brain function.

## 3 Neurophysiological and behavioral effects of tSMS

### 3.1 Effects of motor evoked potentials (MEPs)

Like other NIBS techniques, the effects of tSMS have been predominantly studied over the motor cortex, using TMS-evoked motor potentials (MEP) as a physiological marker. Multiple studies have consistently shown that tSMS over the primary motor cortex (M1) suppresses corticospinal excitability, reflected in reduced MEP amplitude.

The first study demonstrating tSMS effects in humans (Oliviero et al., 2011) included four experiments, two of which were single-blind and two sham-controlled, double-blind. They showed that 10 min of M1-tSMS (45 × 30 mm N45 magnet) reduced MEP amplitude for about 6 min post-stimulation, whereas smaller magnets or sham had no effect. Silbert et al. (2013) confirmed MEP suppression in a sham-controlled, double-blind, crossover study with 15-minute tSMS using the same magnet. Nojima et al. (2015) used a slightly larger and stronger 50 × 30 mm N50 magnet in a sham-controlled, single-blind, parallel design and found that 20-minute tSMS over the left M1 reduced MEPs, and increased resting motor threshold and SICI. Dileone et al. (2018) confirmed increased SICI and reported reduced short-interval intracortical facilitation (SICF) after 10-minute tSMS in a sham-controlled, double-blind, parallel study. In a follow-up parallel study, Nojima et al. (2016) showed that 5-minute tSMS alone was insufficient but effectively suppressed MEPs when combined with peripheral nerve stimulation. Arias et al. (2017) used a sham-controlled, crossover design to demonstrate that 20-minute tSMS (45 × 30 mm N45 magnet) reduced MEPs and further showed that sensorimotor integration measures such as short/long afferent inhibition remained unchanged. Davila-Pérez et al. (2019) used a sham-controlled, crossover design to show that 15-minute tSMS (38.1 × 38.1 mm N42 magnet) reduced MEPs and increased SICI and LICI, but only with specific TMS pulse configurations, suggesting circuit-specific effects.

Interestingly, Kufner et al. (2017), in a sham-controlled, single-blind design, did not observe MEP suppression with 10-minute tSMS (45 × 30 mm N45 magnet). At the time, it was suggested that concurrent cognitive engagement during stimulation might have contributed to the absence of effects (Foffani and Dileone, 2017). However, subsequent studies using within-subject designs that explicitly controlled for cognitive engagement did not support this explanation, as they also failed to detect modulation of corticospinal excitability following short-duration tSMS (Lorenz et al., 2020). Similar null findings have been reported in more recent work applying 20-minute stimulation across both hemispheres, which failed to detect changes in MEPs or paired-pulse measures (Lorenz et al., 2026). In clinical populations, a placebo-controlled study in ALS patients also reported no short-term changes in corticospinal excitability following tSMS (Stephani et al., 2024), suggesting that disease-related factors and pharmacological treatment may further influence responsiveness. Notably, several of these studies employed neuronavigation to ensure precise and stable TMS coil positioning, whereas many earlier studies reporting positive effects relied on conventional hotspot-based approaches. Based on this pattern, it has been proposed that highly focal and consistent targeting might reduce the likelihood of capturing tSMS effects (Lorenz et al., 2026). However, this interpretation should be considered with caution, as positive effects have also been demonstrated in studies using neuronavigation (Pagge et al., 2025, Pagge et al., 2024), indicating that coil positioning accuracy alone cannot account for the variability of findings. It is also worth noting that neuronavigation is rarely applied to tSMS itself, as magnet placement typically relies on anatomical landmarks or hotspot-based positioning, and the relatively broad spatial extent of static magnetic fields reduces the need for millimetric targeting. Such discrepancies highlight the sensitivity of tSMS outcomes to contextual and methodological factors. Taken together, available evidence suggests that applying tSMS over M1 for 10 to 20 min can induce a transient suppression of corticospinal excitability, although effects appear to be variable across studies and may depend on specific experimental conditions, and are likely accompanied by changes in intracortical excitability.

**Fig. 2:**
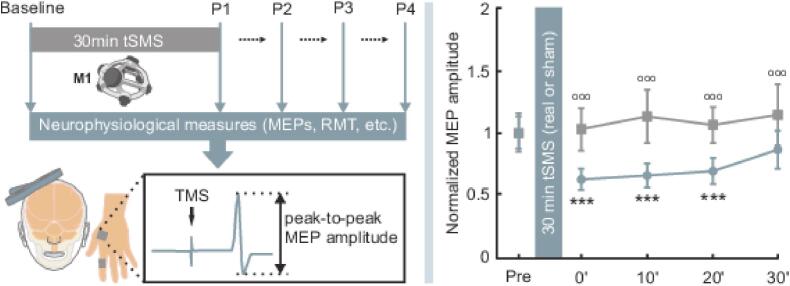
Experimental design and results, when M1-tSMS is applied 30 min on the primary motor cortex (M1). (Left panel) Representative experimental protocol applying M1-tSMS for 30 min measuring motor evoked potentials (MEPs), resting motor threshold (RMT), and related measures using transcranial magnetic stimulation (TMS) before and after tSMS. Motor responses were recorded from the first dorsal interosseous. (Right panel) Average changes in corticospinal excitability induced by 30 min of real or sham tSMS, expressed as MEP amplitude normalized to the group-average baseline. Error bars represent standard errors of the mean. *Comparisons vs. Pre (Baseline); °comparisons real vs. sham at the same time point. 3 symbols: p < 0.001, 2 symbols: 0.001 < p < 0.01; 1 symbol: 0.01 < p0.05. Modified from Dileone et al. (2018), with permission.

Long-lasting effects of tSMS are especially important for its potential therapeutic use. Thus, Dileone et al. (2018) applied 30-minute tSMS (45 × 30 mm N45 magnet) over M1 in a series of sham-controlled, double-blind and single-blind studies. They found MEP suppression lasting at least 30 min post-stimulation (Fig. 2), along with a long-term decrease in SICI and an increase in SICF—suggesting a shift in intracortical dynamics not observed with shorter durations. While some studies using similar stimulation durations have reported less consistent effects, for example Hamel et al. (2020) did not observe modulation of corticospinal excitability following 30-minute tSMS, these findings may depend on specific experimental conditions, including the cortical hemisphere targeted.

In line with this variability, more recent studies have further explored how stimulation parameters and cortical targets influence the magnitude and consistency of tSMS effects. Pagge et al. (2025), in an open-label pilot experiment, compared 30-minute tSMS over right vs. left M1 (60 × 30 mm N52 magnet). They found stronger and more consistent MEP suppression after right M1 stimulation, suggesting hemispheric differences in responsiveness, possibly linked to handedness. In another open-label pilot study, Pagge et al. (2024) applied 30-minute tSMS to the SMA (60 × 30 mm N52 magnet) and also observed MEP suppression at M1, pointing to the possibility of modulating broader motor networks through connected regions.

Long-lasting effects of tSMS are especially important for its potential therapeutic use. Thus, Dileone et al. (2018) applied 30-minute tSMS (45 × 30 mm N45 magnet) over M1 in a series of sham-controlled, double-blind and single-blind studies. They found MEP suppression lasting at least 30 min post-stimulation (Fig. 2), along with a long-term decrease in SICI and an increase in SICF—suggesting a shift in intracortical dynamics not observed with shorter durations. While some studies using similar stimulation durations have reported less consistent effects, for example Hamel et al. (2020) did not observe modulation of corticospinal excitability following 30-minute tSMS, these findings may depend on specific experimental conditions, including the cortical hemisphere targeted.

Together, these findings confirm that tSMS can reliably reduce corticospinal excitability in healthy individuals, with stimulation duration, cortical target, and individual factors such as hemispheric dominance shaping the magnitude and persistence of its effects.

### 3.2 Impact on sensory-evoked responses

In addition to modulating motor output, tSMS has been shown to influence cortical sensory processing, particularly as measured by somatosensory evoked potentials (SEPs). Across studies, short applications of tSMS generally suppress early SEP components, suggesting a transient reduction in sensory cortical excitability.

In a sham-controlled, double-blind, crossover study, Kirimoto et al. (2014) applied 10–15 min of tSMS (50 × 30 mm N45 magnet) over the left sensorimotor cortex (C3). They observed a significant post-stimulation reduction in the N20 component amplitude elicited by right median nerve stimulation, but only when SEPs were recorded after the intervention, not during—highlighting the importance of network state in mediating neuroplastic changes. In a follow-up study, the same team compared stimulation over the left motor cortex vs. the SMA using the same magnet and a 15-minute protocol. Only M1 stimulation reduced the N33 component at left parietal cortex (C3′), indicating region-specific responsiveness (Kirimoto et al., 2016). Further supporting the spatial specificity of SEP effects, Carrasco-López et al. (2017) conducted two sham-controlled, double-blind, crossover studies applying 20-minute tSMS (60 × 30 mm N40 magnet) over C3′. They observed selectively enhanced alpha-band oscillatory power, without producing significant changes in early cortical components of SEPs that reflect bottom-up thalamocortical input. By contrast, Kufner et al. (2017), applying a 45 × 30 mm N45 magnet for 10 min 2.25 cm posterior to the motor hotspot, found no SEP changes—emphasizing how small variations in magnet placement can affect outcomes.

Kirimoto et al. (2018) extended this line of work to cortical nociceptive processing. In a sham-controlled, double-blind, crossover, they applied 15-minute tSMS (50 × 30 mm N45 magnet) over either M1 or the primary somatosensory cortex. Only M1 stimulation suppressed potentials evoked by intra-epidermal electrical stimulation, which selectively activates A-delta and C nociceptive fibers —suggesting that tSMS may also modulate cortical processing of pain.

Further supporting the applicability of tSMS to sensory systems, Heimrath et al. (2020) applied 20-minute tSMS (60 × 30 mm N40 magnet) over the auditory cortex in a sham-controlled, single-blind, crossover study. Only left-hemisphere stimulation reduced the N1 component of auditory evoked potentials, suggesting lateralized effects. In contrast, Azcona Ganuza and Alegre (2022), applying 30-minute tSMS (45 × 30 mm N45 magnet) over the left auditory cortex in a sham-controlled, crossover study, reported no changes in auditory evoked potentials or gamma-band responses—suggesting tSMS may have limited efficacy in modulating auditory cortex in the absence of hyperexcitability.

Collectively, these studies show that tSMS can transiently suppress early cortical responses across multiple sensory modalities, including tactile, nociceptive, and auditory inputs. The consistent findings across controlled studies highlight evoked potentials as reliable markers of tSMS-induced changes in cortical excitability.

### 3.3 Changes in cortical excitability measured by EEG and fMRI

Beyond responses to external stimuli, the effects of tSMS have also been explored on ongoing spontaneous brain activity using electroencephalography (EEG) and resting-state functional MRI (fMRI), providing insight into both local excitability and broader network modulation.

In the visual cortex, Gonzalez-Rosa et al. (2015) applied 10-minute tSMS (60 × 30 mm N40 magnet) and reported increased alpha power during stimulation. This frequency-specific, focal effect was confirmed in two independent experiments—one single-blind, sham-controlled, crossover design, and one double-blind, parallel design. Carrasco-López et al. (2017) reported a similar alpha enhancement in the somatosensory cortex following 20-minute tSMS with the same magnet, showing site- and duration-dependent effects. In contrast, a study targeting the dorsolateral prefrontal cortex (DLPFC) found no increase in local alpha oscillations, likely due to the frontal cortex’s low alpha dominance. Sheffield et al. (2019) applied 10-minute tSMS (63.4 × 25.4 mm N52 magnet) over left or right DLPFC (sham-controlled, double-blind, crossover design) and observed oscillatory modulation in remote regions, suggesting network-level rather than local effects.

Resting-state fMRI studies corroborate these findings. Pineda-Pardo et al. (2019), using 30-minute tSMS over the SMA (45 × 30 mm N45 magnet) in a sham-controlled, double-blind, crossover study, reported increased local activity in the SMA and enhanced functional connectivity with paracentral and frontotemporal areas, including the inferior frontal gyrus. In contrast, Soto-León et al. (2022) used a 10-minute tSMS protocol over left M1 (45 × 30 mm N45 magnet) and reported transient reductions in functional connectivity within the sensorimotor network, with no significant changes in the default mode or visual networks. Building on this, Caballero-Insaurriaga et al. (2023) developed and applied the ISAAC framework to disambiguate functional connectivity between regions from local activity within regions in resting-state fMRI. Using this method, they showed that 30-minute SMA-tSMS (60 × 30 mm N52 magnet) increased activity in the SMA, adjacent sensorimotor cortex, and motor striatum. Model-based analysis indicated that striatal modulation was primarily driven by shared activity with cortical regions—pointing to corticostriatal modulation by tSMS.

Shibata et al. (2021) applied 20-minute tSMS (50 × 30 mm N50 magnet) over the left M1 and found increased theta power at the stimulation site, along with enhanced theta-band functional coupling between left M1 and parietal regions. These findings point to local modulation and altered network-level connectivity, particularly in the theta band. Building on this, Shibata et al. (2024) reported that the same tSMS protocol suppressed the P30 component and enhanced the N60 contralaterally, suggesting both local and interhemispheric modulation of evoked responses. Alpha-band oscillatory activity was also suppressed bilaterally, further supporting the ability of tSMS to affect cortical dynamics beyond the stimulation site.

Altogether, EEG and fMRI studies demonstrate that tSMS can modulate both spontaneous and evoked neural activity, producing localized changes in cortical rhythms and broader adjustments in functional connectivity.

### 3.4 Effects on behavioral performance

Although initially focused on physiological modulation, tSMS has also been shown to influence behavior, spanning sensory perception, cognition, and motor control. These effects provide causal insights into brain–behavior relationships and support potential therapeutic applications.

In the visual domain, Gonzalez-Rosa et al. (2015) applied 10-minute tSMS (60 × 30 mm N40 magnet) over the occipital cortex and observed slowed reaction times in a demanding visual search task, with effects linked to increased alpha oscillations. The behavioral effect was replicated in a second experiment with EEG recordings, where higher alpha predicted slower responses, suggesting reduced top-down modulation. Lozano-Soto et al. (2017) showed that a similar tSMS protocol reduced discomfort from high-intensity light stimuli, consistent with an inhibitory effect on visual cortex excitability. More recently, Takami et al. (2024) applied 30-minute tSMS (60 × 30 mm N52 magnet) over the left hMT+ (human MT + complex, involved in motion processing) and found reduced impaired visual motion discrimination, with subsequent work showing reduced visuomotor spatial accuracy during continuous tracking (Takami et al., 2025), especially for contralateral stimuli indicating that tSMS can modulate perceptual processing in a spatially specific manner, consistent with the functional organization of visual areas (Grill-Spector and Weiner, 2014).

In the auditory domain, Heimrath et al. (2020) applied 20-minute tSMS (60 × 30 mm N40 magnet) over auditory cortex in a sham-controlled, single-blind crossover study. Only left-sided stimulation reduced right ear advantage in a dichotic listening task, suggesting lateralized modulation of auditory processing. Meanwhile in the somatosensory system, Carrasco-López et al. (2017) showed enhanced detection of near-threshold tactile stimuli after 20-minute tSMS over the parietal cortex (60 × 30 mm N40 magnet) in two sham-controlled, double-blind studies. This paradoxical enhancement may reflect reduced habituation due to alpha-band modulation, and highlights a dissociation between evoked potentials and perceptual sensitivity.

Cognitive effects of tSMS have been explored in a range of tasks targeting the DLPFC and SMA. Chen et al. (2021) applied 26-minute tSMS over the left DLPFC (50 × 30 mm N48 magnet) in a sham-controlled, within-subject, crossover design. They found impaired working memory and prolonged N2 event-related potential components, indicating that tSMS modulates DLPFC activity. Soto-León et al. (2023) used 20-minute tSMS (60 × 30 mm N52 magnet) in a double-blind, crossover design and observed increased randomness in a number generation task, interpreted as enhanced inhibitory control. In contrast, Watanabe et al. (2021) found no effects on a Go/NoGo task using 30-minute tSMS (50 × 30 mm N48 magnet) over left or right DLPFC, although their follow-up study (Watanabe et al., 2023) reported impaired 2-back task performance and increased beta synchronization after left DLPFC stimulation. These findings suggest that tSMS effects depend on task and stimulation parameters.

Studies targeting the SMA highlight its role in decision-making and motor control. In a previously mentioned study, the modulation of decision-making parameters across reaction time tasks was found (Pineda-Pardo et al., 2019). Conversely, Guida et al. (2023), using a crossover design, found no effect (45 × 30 mm N45 magnet) on automatic or voluntary response inhibition in a Go/NoGo learning task or stop-signal task. Carpio et al. (2024) applied 30-minute tSMS (45 × 30 mm N45 magnet) over the SMA in a double-blind, sham-controlled crossover study and reported increased decision thresholds in a virtual reality Go/NoGo task involving close-proximity stimuli. Tsuru et al. (2020) used 20-minute tSMS (50 × 30 mm N48 magnet) over the SMA and found refined postural timing in anticipatory adjustments during shoulder flexion, supporting the SMA’s involvement in preparatory and executive motor functions.

Two studies have explored how tSMS over the motor cortex may influence motor learning. Nakagawa et al. (2019) applied 15-minute tSMS (50 × 30 mm N50 magnet) over either the left or right M1 in a sham-controlled, single-blind, crossover design. While voluntary force output was unchanged, tSMS increased the absolute error in a pinch force task, suggesting reduced precision, potentially due to altered somatosensory feedback. In contrast, Nojima et al. (2019) delivered 10-minute tSMS over the right M1 during training in a serial reaction time task, using a single-blind, parallel-group design. They found improved offline motor learning 24 h later, indicating enhanced memory consolidation. These findings suggest that tSMS over M1 can differentially influence motor precision and learning, depending on task context and timing.

Together, these studies demonstrate that tSMS can modulate a wide range of behavioral functions, from basic sensory detection to higher-order cognitive and motor control. Importantly, most behavioral effects appear to be modest and task-dependent, emphasizing the need for optimized protocols and larger sample sizes to fully characterize the scope of tSMS as a behavioral neuromodulation tool.

## 4 Neurological disorders and home-based applications

Building on its favorable safety profile, simplicity, and growing evidence of physiological efficacy, tSMS has begun to attract attention as a candidate for clinical application in neurological disorders. Particularly compelling is its suitability for home-based use—enabling patients to perform regular neuromodulation sessions without the logistical and financial burden of in-clinic visits. This section reviews current evidence on tSMS in clinical populations—focusing on home-based use in Parkinson’s disease (PD), stroke, and amyotrophic lateral sclerosis (ALS)—and outlines key opportunities and challenges for broader clinical translation.

### 4.1 Movement disorders: home-based tSMS for hyperkinetic manifestations

Levodopa-induced dyskinesias (LID) are a frequent and often disabling complication in PD, particularly after long-term dopaminergic treatment. LID represents a hyperkinetic state, and low-frequency (inhibitory) rTMS protocols targeting motor areas have been explored to counteract the underlying cortical hyperexcitability (Wu et al., 2021). This therapeutic rationale is supported by neurophysiological evidence showing reduced intracortical inhibition across all stages of PD, including in newly diagnosed and less affected hemispheres (Ammann et al., 2020), suggesting early and widespread cortical disinhibition.

In line with this pathophysiological framework, a randomized, double-blind, sham-controlled trial provided the first clinical evidence using tSMS (Dileone et al., 2022). Fifty PD patients received either real or sham tSMS over M1 contralateral to the more affected side, with one 30-minute session per day over nine non-consecutive days. Participants could choose home or hospital delivery; notably, all but one opted for home-based administration, underscoring its practicality. Objectively, both groups showed a moderate improvement in LID severity as assessed by the Unified Dyskinesia Rating Scale, but there was also moderate evidence supporting the absence of a difference between real and sham groups. Motor symptoms remained unchanged. In contrast, subjective impressions captured via the Patient’s Global Rating of Change significantly favored real tSMS, suggesting that patients perceived greater benefit despite the lack of measurable clinical difference. These findings suggest that while the brief tSMS course may not yield objective improvement, it can positively influence patients' perception of their symptoms. One hypothesis proposed by the authors is that tSMS modulates brain networks linked to bodily awareness or sense of agency, rather than directly suppressing involuntary movements. Alternatively, the limited number of sessions may have been insufficient to reveal group differences—longer protocols, as used in other NIBS studies, might yield more robust effects. Importantly, tSMS was well tolerated, with no serious adverse events. Minor effects—such as transient dizziness, headache, or a mild bruise in one frail patient—were self-limiting and occurred with equal frequency in both groups. A custom helmet ensured accurate positioning, contributing to high adherence and safety.

Together, these findings established the clinical feasibility, safety, and patient acceptability of tSMS in hyperkinetic movement disorders, particularly in the context of repeated and potentially home-based interventions.

Consistent with this framework, the clinical applicability of tSMS has more recently been extended beyond PD. In a randomized, double-blind pilot study in essential tremor, a single 30-minute session of unilateral M1 tSMS, delivered in a supervised clinical setting, induced a moderate short-term reduction of postural and rest tremor, with bilateral effects and no significant safety concerns (Urso et al., 2025), further supporting the potential of tSMS as a portable, well-tolerated neuromodulatory approach in hyperkinetic conditions and as a promising candidate for future home-based applications.

### 4.2 Stroke rehabilitation: tSMS and motor recovery

Stroke often results in motor deficits due to damage in one hemisphere, with the intact hemisphere exerting excessive inhibitory influence on the lesioned side (maladaptive interhemispheric inhibition) (Murase et al., 2004). Neuromodulation strategies such as low-frequency rTMS or cathodal tDCS have sought to downregulate excitability in the contralesional motor cortex to restore interhemispheric equilibrium and facilitate motor rehabilitation (Lefaucheur et al., 2020, Lefaucheur et al., 2017). Building on this rationale, Takamatsu et al. (2021) demonstrated in healthy participants that tSMS applied over the left M1 not only suppressed local excitability but also enhanced excitability in the contralateral M1. This bidirectional modulation was associated with a reduction in interhemispheric inhibition from left to right M1, supporting the use of tSMS to rebalance interhemispheric dynamics through remote network effects.

One important advantage in the context of neurorehabilitation is that tSMS does not induce electrical currents, avoiding common stimulation-related risks such as pain or seizures. These properties facilitate its integration into standard therapy sessions and may support future use in less supervised environments. To date, investigations in stroke patients have been primarily limited to clinical research settings, where tSMS is applied alongside conventional rehabilitation to assess its feasibility and therapeutic potential.

The first clinical application of this approach was reported by Shimomura et al. (2023) in a randomized, sham-controlled, double-blind crossover trial in subacute stroke. Twenty patients with hemiparesis (≤6 months post-stroke) received real or sham tSMS over the contralesional M1 for 20 min prior to daily occupational therapy, in two separate two-week phases. Real tSMS led to significantly greater improvements in paretic hand dexterity, as measured by a timed hand-finger movement task. Neurophysiological data confirmed reduced excitability in the contralesional M1 and increased excitability in the ipsilesional M1, indicating effective modulation of interhemispheric balance. All patients completed the protocol without adverse events, and treatment was well tolerated. Although delivered in a supervised clinical setting, the straightforward application of tSMS suggests that future home-based use may be feasible. Together, these findings provide the first evidence that tSMS can enhance motor recovery after stroke by promoting cortical reorganization, encouraging further trials to assess long-term efficacy, scalability, and optimal stimulation parameters.

### 4.3 Amyotrophic lateral sclerosis: home-based tSMS and cortical hyperexcitability

ALS is a progressive neurodegenerative disease marked by upper and lower motor neuron loss. A consistent early finding is cortical hyperexcitability—excess motor cortex output thought to drive downstream motor neuron degeneration (Geevasinga et al., 2016). This has motivated efforts to develop neuromodulatory interventions that reduce cortical excitability as a strategy to slow disease progression. Non-invasive techniques such as rTMS and tDCS have shown mixed results—ranging from inconclusive to more encouraging findings (Benussi et al., 2023, Fang and He, 2013, Guo et al., 2011). Moreover, invasive approaches like epidural motor cortex stimulation have shown potential in isolated ALS cases, including one remarkable report of prolonged survival exceeding 15 years (Di Lazzaro et al., 2017).

Given the need for frequent, long-term intervention in ALS, tSMS has emerged as a promising candidate especially due to its home-use profile. An open label pilot study by Di Lazzaro et al. (2021) demonstrated the feasibility and safety of self-administered home-based tSMS in two patients with rapidly progressive ALS, who applied the helmet three times daily over both M1 cortices for several months. As a result, both patients showed a *“dramatic and prolonged reduction in disease progression”*. Although uncontrolled, this pilot raised the exciting possibility that tSMS could slow ALS progression.

This promising finding led to a bicentric randomized, double-blind placebo-controlled phase 2 trial, where 40 ALS patients received either real or sham tSMS over both motor cortices three times daily for six months at home (Di Lazzaro et al., 2024). Over this period, there was no significant difference between the real and sham groups in progression measured by the Amyotrophic Lateral Sclerosis Functional Rating Scale – Revised (p = 0.83), nor in corticospinal excitability. Similarly, a recent study in ALS patients reported no changes in cortical excitability following a single 10-minute tSMS session (Stephani et al., 2024). However, during the 18-month follow-up from Di Lazzaro et al. (2024), a significant difference in tracheostomy-free survival emerged: patients who had received real tSMS had a 73% lower risk of death or tracheostomy compared to sham (hazard ratio = 0.27, *p* = 0.019). This finding suggests that tSMS might exert a delayed or cumulative neuroprotective effect that influences long-term survival, even if not immediately reflected in functional scores. From a safety and feasibility standpoint, the trial was highly encouraging. Eighty percent of participants completed the full protocol, and no serious adverse events were attributed to tSMS. Side effects were mild and infrequent, including occasional scalp discomfort or fatigue. Given the burden of frequent clinic visits in ALS, the high adherence and safety of daily home-based stimulation highlight the practicality of this approach even in a population with advanced disability. While tSMS did not demonstrate short-term efficacy in this trial, the survival signal observed in the long-term follow-up warrants further investigation. Longer treatment durations or combination strategies with pharmacological agents may be needed to amplify therapeutic effects.

From a practical perspective, the weight of the stimulation system represents an important consideration for long-term use, particularly in clinical populations with progressive motor impairment. In the phase 2 trial, the combined weight of the magnet and helmet system was approximately 2 kg, and patients were instructed to remain at rest during stimulation to maintain stable positioning. Although overall adherence was high, some participants reported mild cervical discomfort attributed to the device weight, which in one case led to treatment discontinuation. Notably, disease progression—especially involving neck and bulbar muscles—may further influence tolerability over time. In both the pilot and phase 2 studies, patients commonly used external support (e.g., a headrest) during stimulation sessions, which likely facilitated comfortable positioning and helped accommodate the weight of the device. These findings suggest that, while home-based tSMS is feasible and generally well tolerated, device ergonomics and weight may influence long-term adherence in certain patient groups. Future developments toward lighter and more adaptable wearable systems may further enhance usability and therapeutic potential. Overall, these considerations, together with the encouraging safety and adherence profiles reported to date, support the practicality of long-term, home-delivered tSMS and its further investigation as part of a multimodal strategy in ALS.

## 5 Development of tSMS

### 5.1 Current and future improvements in magnet-based stimulation technology

Early implementations of tSMS used simple cylindrical neodymium magnets (e.g., N45 grade), but the field has since evolved toward stronger materials like N52, allowing for higher B-fields and steeper field gradients. Standardization efforts have focused on magnet size, shape, and material composition to ensure reproducibility of stimulation effects. Notably, biological tissue has minimal impact on the static field, simplifying dosing and modeling (Rivadulla et al., 2014).

To improve spatial targeting and depth penetration, researchers have developed high-gradient or curved magnets and multi-magnet arrays. The “SHIN jiba” system, using a fixed triangular array of three N52 magnets, enhances field strength and cortical coverage (Shibata et al., 2022), and reliably suppressed MEPs over M1. Using this setup, Matsumoto et al. (2023) applied bilateral tSMS over the motor association cortex in a sham-controlled, within-subject study, finding increased reaction time variability—an effect absent with single magnets or unilateral stimulation. Tomita et al. (2024) used the triple-array system for 15-minute tSMS in individuals with high social anxiety, observing reduced self-focused attention during a verbal task. These results support the utility of stronger, broader fields for modulating higher-order associative cortices—and potentially deeper structures. In support of this, Hoshi et al. (2025) applied “SHIN jiba” tSMS over the lumbar spine in a single-blind, crossover study and found significant suppression of spinal H-reflexes immediately after stimulation, with no SEP changes, indicating selective spinal inhibition and potential for therapeutic use in conditions involving hyperexcitability.

Extending this approach, recent numerical modeling combined with experimental characterization of magnetic fields has systematically explored how multi-magnet configurations can be optimized to achieve broader cortical coverage and deeper field penetration. Using realistic pediatric head models, Paz et al. (2025) demonstrated that arrays of up to ten neodymium magnets can generate magnetic flux densities within the proposed therapeutic range (>0.10 T) across more than 70% of the cortical surface and reach deep brain regions near the base of the brain, highlighting the importance of magnet number, orientation, and interaction effects for future tSMS device design.

Looking ahead, next-generation tSMS technology will likely prioritize improved targeting and ease of use. One promising avenue is the development of flexible, wearable systems—such as custom-fitted helmets or magnetized caps aligned to anatomical landmarks—for accurate, repeatable stimulation with minimal effort, particularly suited for home-based protocols in conditions like stroke or ALS. Arrays of smaller magnets could also be designed to simultaneously target bilateral cortical areas, enabling more complex interventions like interhemispheric rebalancing after stroke. Also, mechanically shifting the magnet—such as through slight oscillation or sliding along a track—could potentially reduce habituation and engage broader neural regions, offering added benefits while preserving the simplicity of tSMS. Advances in magnet materials may also yield smaller yet stronger magnets that increase depth without compromising safety or usability. Still, higher field strength must be balanced against safety standards and regulatory limits. To ensure reliability, future tSMS devices may incorporate tools to verify magnet integrity—such as field strength and polarity checks—as part of routine standardization protocols.

tSMS is also emerging as a promising element in multimodal neuromodulation. Micera and Foffani (2024) argue that no single NIBS technique can fully address the complexity of brain function and advocate for combining physical modalities—electrical, magnetic, acoustic, or optical—in personalized, synergistic ways. tSMS offers advantages such as portability, long-term compatibility, and minimal tissue distortion, making it well-suited for integration. While its depth and spatial precision are limited, these features may complement more focal or deeper techniques (e.g., ultrasound or optogenetics) within tailored multimodal intervention strategies.

Finally, integration with real-time monitoring tools such as EEG and fMRI is enabling more dynamic assessment of tSMS effects and individualized targeting (Shibata et al., 2024, Soto-León et al., 2022). Computational modeling and AI-based approaches are also expected to help optimize stimulation protocols for specific pathophysiological states. Together, these advancements are positioning tSMS not just as a standalone technique, but as a scalable and flexible tool in the broader landscape of precision neuromodulation.

### 5.2 Home-based tSMS, patient compliance and digital integration

tSMS is particularly suited for wearable and home-based applications due to its passive nature and lack of powered components. Recent years have seen the development of ergonomic, user-friendly devices aimed at standardizing tSMS delivery. A key example is the MAGm series by Neurek SL (Fig. 1), which has evolved across multiple versions to accommodate various head sizes and cortical targets: from the motor and visual cortices (MAGmv1.0 and 1.1) to the dorsolateral prefrontal and parietal cortices (MAGdpv1.1), and the SMA (MAGsv1.1). This evolution reflects increasing interest in adapting tSMS for both research and home-based clinical use, with improvements in precision, comfort, and reproducibility. Practical advantages—including simplicity, safety, affordability, and ease of use without specialized staff—make tSMS an attractive option for clinical translation (Nojima et al., 2020). Ergonomic helmet-based systems are especially promising for chronic use in rehabilitation or preventive neuromodulation. However, challenges remain: anatomical variability, particularly scalp-to-cortex distance, influences magnetic field strength at the target. Modeling studies confirm that field intensity decays exponentially with distance, underscoring the need for individualized setups, potentially guided by neuroimaging or computational modeling (Viudes-Sarrion et al., 2021).

Home-based tSMS is particularly advantageous for protocols requiring repeated or daily sessions, easing the burden of regular clinic visits. To support safe and unsupervised use, modern helmet systems incorporate fixed cylindrical holders to ensure consistent magnet placement and are stored in protective cases that attenuate external magnetic exposure, minimizing accidental attraction to ferromagnetic surfaces. Before initiating treatment, patients and caregivers typically undergo brief, structured training at the hospital, covering correct helmet positioning, warnings about the strength of the magnets, and safety precautions (e.g., avoiding proximity to metal objects, electronic devices, children, and individuals with pacemakers or metallic implants). These measures help reduce risk and support patient confidence when handling the device at home.

**Fig. 3:**
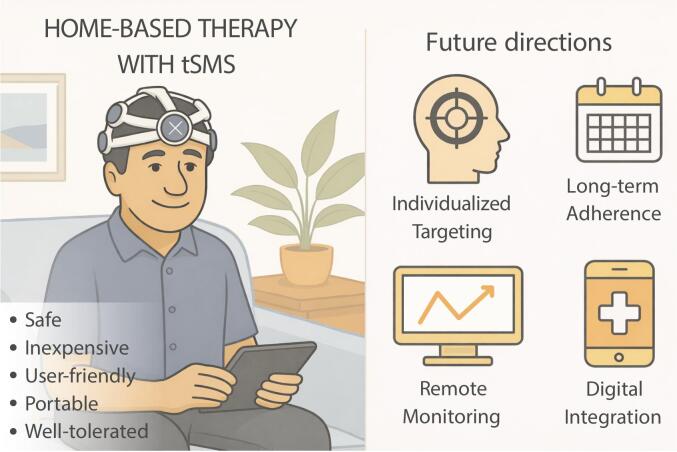
Transcranial static magnetic field stimulation (tSMS) for home use: Current benefits and future innovations.

Ergonomic features that accommodate various head shapes and enable easy self-application—without specialized training—are central to current development, enhancing usability and patient adherence. In the PD dyskinesia trial, nearly all participants opted for home application and completed all nine sessions (Dileone et al., 2022). In the 6-month ALS trial, adherence reached ∼ 80% over a prolonged period (Di Lazzaro et al., 2024), a strong result given the severity of the condition. Additionally, the absence of perceptible sensation helps maintain successful blinding and improves comfort. Still, sustaining long-term adherence outside structured trials may be challenging; chronic applications may benefit from integration with telehealth tools or patient support systems (Fig. 3).

Implementing home-based tSMS comes with challenges, particularly around remote monitoring—ensuring patients use the device correctly and as prescribed. While current studies rely on patient diaries or periodic check-ins, future systems are likely to integrate digital tools (Chirra et al., 2019, Espay et al., 2016). tSMS is well-suited for incorporation into digital health platforms, where companion smartphone apps could provide reminders, video-guided placement instructions, and magnet position verification via onboard sensors (e.g., magnetometer). These apps could also collect patient-reported outcomes through symptom check-ins or short surveys after each session. Wearable devices, like smartwatches, might track physiological data—such as tremor in PD or activity levels in ALS—enabling temporal correlations with tSMS use (van Unnik et al., 2025). Additional tools, such as home spirometry or speech assessments (Neumann et al., 2024), could monitor disease progression more frequently and offer new clinical endpoints. These digital features could integrate with telemedicine platforms to allow timely clinician follow-up if data indicate poor adherence or limited treatment response (Pathak et al., 2021, Thankathuraipandian et al., 2024).

Digital integration paves the way for personalized, data-driven optimization of tSMS. As more patients use the technology and contribute outcome data, algorithms—potentially AI-based—could identify usage patterns linked to better results. Some individuals might respond better to multiple short sessions, others to varied stimulation targets. Closed-loop systems could emerge, adjusting stimulation parameters weekly based on individual response (Belkacem et al., 2023, Zrenner et al., 2016). At the systems level, cloud-connected tSMS devices would enable large-scale monitoring, supporting real-world studies on efficacy and best practices while preserving privacy through de-identified datasets. Additionally, tSMS could be combined with remote interventions like cognitive training or rehab games to boost neuroplasticity via multimodal stimulation.

Together, these advances position tSMS as a strong candidate for home-based neuromodulation, combining technical simplicity with growing digital integration. While challenges remain—particularly around individualized targeting and long-term adherence—the emergence of ergonomic devices, remote monitoring tools, and data-driven personalization strategies support its feasibility for widespread clinical deployment. Continued innovation at the interface of bioengineering, digital health, and neuroscience will be key to unlocking the full potential of tSMS in real-world therapeutic settings.

### 5.3 Safety considerations and regulatory perspectives

The safety profile of tSMS is highly favorable, supporting its suitability for clinical and home-based use. Human studies confirm its tolerability; for example, a 2-hour application over the occipital cortex with a 60 × 30 mm N40 magnet showed no adverse effects on cognition, neurophysiological markers, or comfort (Oliviero et al., 2015). Biomarkers like neuron-specific enolase remained stable, and the observed S100 protein decrease—also seen with sham—was considered non-specific. Preclinical studies using stronger (∼300mT) or prolonged exposures (up to 24 h) have reported cellular effects, such as reduced neuroblastoma cell viability (Medeiros et al., 2020) and oxidative imbalance in rat astrocytes (da Costa et al., 2021). These findings highlight the need to define safe parameters, though the conditions far exceed standard human tSMS protocols.

Most tSMS protocols generate magnetic fields well below the safety thresholds set by the International Commission on Non-Ionizing Radiation Protection (ICNIRP), which allows up to 2 T for the head and trunk in occupational settings. At cortex-relevant distances (∼2 cm), tSMS typically delivers under 200 mT (Park et al., 2019, Rivadulla et al., 2014, Tharayil et al., 2018). Even intensive protocols—like three daily sessions totaling 2 h—remain far below these limits (Di Lazzaro et al., 2024, 2021). Decades of MRI use provides further reassurance: static fields up to 7 T, and even 8 T in research, have shown no clinically significant changes in heart rate, core body temperature, or electrocardiograms (Chakeres et al., 2003, Formica and Silvestri, 2004, Kangarlu et al., 2003). Still, caution is advised for individuals with implanted magnetic or metallic devices (e.g., pacemakers or cochlear implants), and magnets must be handled properly to avoid mechanical hazards. In clinical trials for PD, stroke, and ALS, tSMS has been well tolerated. No major adverse effects have been reported; minor symptoms like anxiety or dizziness occurred equally in sham conditions. One practical issue was slight bruising from helmet pressure in a frail patient, highlighting the importance of ergonomic, lightweight device designs for repeated use.

Formal safety guidelines and clearly defined absolute or relative contraindications for tSMS are still evolving. Given the static nature of the magnetic field, potential safety considerations mainly relate to mechanical forces and interactions with nearby ferromagnetic or electronic implants. Accordingly, caution is advised in individuals with implanted devices such as pacemakers, deep brain stimulators, or cochlear implants, and tSMS should not be applied directly over or in close proximity to these systems. The magnetic fields used in tSMS are substantially weaker than those encountered in MRI, and maintaining an appropriate distance minimizes the risk of device interference or displacement. In addition, basic handling precautions—such as ensuring stable fixation of the magnet, careful placement and removal to avoid minor mechanical injury, and avoiding unintended contact with metallic objects—are recommended. These considerations are particularly relevant for home-based applications, where clear instructions and patient education can further support safe use.

From a regulatory perspective, tSMS is classified as a Class I medical device under the EU Medical Device Regulation (MDR 2017/745), reflecting its passive, low-risk nature. This contrasts with recent changes under Annex XVI (C/2022/8638), which reclassify TMS and tDCS used without a medical indication as Class III—posing significant regulatory hurdles despite strong safety records (Antal et al., 2024). In this context, tSMS offers a more accessible, research-friendly alternative.

However, expanding tSMS to home use raises additional ethical and regulatory considerations. While it empowers patients, especially those with limited mobility, it also requires clear guidance on training, informed consent, and emergency protocols—particularly for individuals with cognitive impairment. Despite its low risk, ongoing monitoring remains important.

Equity of access must also be considered. Although affordable by design, costs could rise if proprietary devices or support services are bundled. Insurance reimbursement may be needed to avoid creating a two-tiered system. Additionally, increased availability raises concerns about off-label or unsupervised use. Clinicians must guide therapy decisions and discourage replacing established treatments with unproven self-administered tSMS. Regulatory frameworks may need to address marketing claims and usage guidelines as commercialization grows.

Finally, long-term safety is still under investigation. While current data are reassuring, the effects of daily tSMS over years use are unknown. Safety surveillance, registries, and long-term studies will be essential, especially if tSMS is considered for vulnerable populations like children or individuals with developmental conditions.

In summary, tSMS combines a highly favorable safety profile with comparatively low regulatory complexity relative to other neuromodulation techniques, positioning it as a promising tool for home-based neuromodulation. However, its future implementation must address ethical, regulatory, and accessibility challenges to ensure safe, equitable, and responsible use. By drawing from frameworks developed for other home-based NIBS interventions, such as tDCS, and maintaining transparent engagement with regulators, clinicians, and patients, the field can establish a solid foundation for the future widespread deployment of tSMS in everyday clinical practice.

### 5.4 Key gaps and unresolved research questions

Despite promising progress, key questions remain regarding the mechanisms, optimal application, and long-term effects of tSMS.

*Mechanisms of action:* While tSMS reliably reduces cortical excitability, its biophysical and neurophysiological mechanisms remain only partially understood. Evidence points to several interacting processes: modulation of ion channel kinetics, enhanced chloride conductance, reversible membrane changes, glial involvement, and possible effects on vascular dynamics or mechanosensitive channels. However, effects on brain oscillations and network dynamics remain unclear, with some studies reporting contralateral or remote modulation. *In vivo* recordings and advanced imaging will be key to understanding local and network-level effects.

*Dose and protocol optimization:* Protocols vary widely in duration (10–30 min), frequency (once to thrice daily), and total length (days to months), but systematic dose–response data are lacking. Critical questions include: What is the minimal effective dose? Is one long session better than multiple short ones? The ALS trial (Di Lazzaro et al., 2024), for instance, showed no short-term benefit but suggested a delayed survival effect—raising the possibility that longer or more intensive regimens may be needed. Optimizing these parameters will require controlled studies with long-term follow-up.

*Predictors of clinical response:* Identifying who benefits most remains a major challenge. Biomarkers such as baseline excitability, EEG patterns, or genetics could help predict outcomes. Future trials should link physiological changes with clinical improvements to guide individualized protocols.

*Comparative effectiveness and combinatorial use:* To position tSMS among other NIBS techniques, comparative studies are needed. While simpler and safer than TMS or tDCS, tSMS may induce weaker or shorter-lasting effects. Head-to-head trials and combination strategies—e.g., tSMS priming before rTMS— could help clarify how best to use each tool.

*Expanding clinical indications:* tSMS has mainly been tested in motor disorders, but its inhibitory effects may extend to conditions like chronic pain, migraine, epilepsy, tinnitus, and depression. The simplicity and safety profile of tSMS make it well suited for exploratory studies in these areas, especially if appropriate targeting strategies are used (e.g., auditory cortex for tinnitus or prefrontal cortex for depression).

*Long-term outcomes:* Sustainability of effects remains unknown. Could long-term tSMS reduce medication needs or slow disease progression? The ALS trial showed improved survival at 18 months, suggesting lasting benefits (Di Lazzaro et al., 2024). Longitudinal studies, real-world registries, and trial extensions will be key to assessing long-term efficacy.

Overall, as tSMS moves closer to clinical implementation, filling these knowledge gaps is essential. Multidisciplinary collaboration—bridging neuroscience, engineering, clinical research, and patient engagement—will be critical. Each new study, regardless of outcome, will help define when, how, and for whom static magnetic fields can be a meaningful therapeutic intervention.

## 6 Conclusions

tSMS has evolved into a promising NIBS technique with unique advantages. Its passive, electricity-free design and strong safety profile make it especially suited for long-term, home-based use. Clinical studies in PD, essential tremor, stroke, and ALS have shown it to be feasible, well-tolerated, and potentially beneficial—ranging from subjective symptom relief to improved motor recovery and possible long-term survival effects. These outcomes support the role of tSMS in self-administered neuromodulation, aligning with trends in telemedicine and patient-centered care. Advances in ergonomic helmet designs, digital integration, and remote monitoring could enable wider adoption and personalized protocols. Its classification as a low-risk device also facilitates clinical translation compared to other NIBS tools. However, further research is needed to clarify mechanisms, optimize protocols, and assess long-term effects. Ethical considerations—such as equitable access and safe use in vulnerable populations—must also be addressed. Overall, tSMS holds strong potential as an accessible, scalable neuromodulation tool. With continued interdisciplinary research, it may become a key component of future self-managed neurotherapies.

## Declaration of competing interest

The authors declare that they have no known competing financial interests or personal relationships that could have appeared to influence the work reported in this paper.
